# Prevalence of resistance markers of artemisinin, partner drugs, and sulfadoxine-pyrimethamine in Nanyumbu and Masasi Districts, Tanzania between 2020 and 2021

**DOI:** 10.1128/aac.01751-24

**Published:** 2025-09-12

**Authors:** Richard O. Mwaiswelo, Bruno P. Mmbando, Frank Chacky, Fabrizio Molteni, Ally Mohamed, Samwel Lazaro, Bushukatale Samuel, Cristina V. Ariani, Sonia Gonçalves, Eleanor Drury, Billy Ngasala

**Affiliations:** 1 Department of Microbiology, Immunology and Parasitology, School of Medicine, Kairuki University 108082, Dar es Salaam, Tanzania; 2 Tanga Research Centre, National Instititute for Medical Research, Tanga, Tanzania; 3 National Malaria Control Programme, Ministry of Health, Dodoma, Tanzania; 4 Department of Medical Parasitology and Entomology, School of Public Health and Social Sciences, Muhimbili University of Health and Allied Sciences 313009, Dar es Salaam, Tanzania; 5 Genomic Surveillance Unit, Wellcome Sanger Institute 47665, Cambridge, United Kingdom; The Children's Hospital of Philadelphia, Philadelphia, Pennsylvania, USA

**Keywords:** prevalence, resistance markers, artemisinin, partner drugs, sulfadoxine-pyrimethamine, Tanzania

## Abstract

Regular monitoring of the emergence and spread of *Plasmodium falciparum* markers of resistance against artemisinin, partner drugs, sulfadoxine, and pyrimethamine is important for the treatment and prevention of malaria in Tanzania. Blood samples were collected from febrile and non-febrile children aged 3 to 59 months in Masasi and Nanyumbu Districts between 2020 and 2021. The samples were subjected to molecular analysis for markers of artemisinin, partner drugs, sulfadoxine, and pyrimethamine resistance, including *Plasmodium falciparum* kelch (*Pfk*) 13 gene, *P. falciparum* chloroquine resistance transporter gene (*Pfcrt*), *P. falciparum* multidrug resistance gene (*Pfmdr*) 1, *P. falciparum* dihydrofolate reductase (*Pfdhfr*), and *P. falciparum* dihydropteroate synthase (*Pfdhps*). A total of 531 blood samples were involved in the analysis. None of the *P. falciparum* isolates analyzed for *Pfk13* carried any of the validated markers of artemisinin resistance. *Pfcrt* CVMNK wild-type haplotype occurred in 88.9% (271/305) of the parasites, and the mutant CVIET haplotype occurred only in 0.7% (2/305). Conversely, the majority of the parasites (24.2% [48/198]) were carrying *Pfmdr1* NFD haplotype, followed by the wild-type haplotype NYD (19.1% (39/198), and the rest were mixed infections. Quintuple mutation **IRN**I-S**GE**AA occurred in 54.4% (62/114), and sextuple mutation **IRN**I-**F**AK**GS** occurred only in 0.9% (1/114) of the parasites. No parasite carried any of the validated markers of artemisinin resistance; however, the prevalence of *Pfcrt* and *Pfmdr1* resistance markers against the partner drugs reached the saturation point. Sextuple *Pfdhfr-Pfdhps* mutations occurred only in one patient; therefore, SP remains efficacious for IPTp in the Districts.

## INTRODUCTION

Tanzania adopted artemisinin-based combination therapy (ACT) as first-line treatment for uncomplicated malaria in 2006 (1). Artemether-lumefantrine (AL) was the first ACT to be adopted, followed by dihydroartemisinin-piperaquine (DHPQ) as alternative first-line, and in 2020, artesunate-amodiaquine (ASAQ) was also adopted (1–3). In Tanzania, ACT has remained efficacious after nearly two decades of its widescale use (4–9). However, the ACT’s efficacy has declined in parts of the world due to the presence of a partial artemisinin resistance (10–16). The resistance is conferred by a point mutation in a *kelch* propeller gene located in chromosome 13 (*k13*) of the *Plasmodium falciparum* parasite (12, 17). The *Pfk13* mutation was first detected in 2013 along the Myanmar-Cambodia border region (17), and since then, it has spread to other countries in the Great Mekong subregion (18, 19). Recently, indigenous *Pfk13* 441L, 469Y/F, 561H, 622I, and 675V mutations were detected in East Africa (13, 16, 20). In Tanzania, *Pfk13* 561H has been detected in five regions, covering all the geographical zones of the country (21–23). Artemisinin resistance in Southeast Asia is associated with the prolonged parasite clearance and increased risks of the partner drug resistance development, treatment failure, gametocyte carriage, and transmission of the infection (18, 24, 25).

The ACT’s cure rate is determined by the long-acting partner drug (26). In Africa, ACT has remained efficacious despite the emergence of artemisinin resistance, probably because the partner drugs are still efficacious (8, 9, 23, 27–30). Different markers of resistance have, however, been linked with parasite tolerance/resistance against the partner drugs. Wild-type *P. falciparum multidrug resistance gene* (*Pfmdr*) 1 N86 and D1246, and *P. falciparum chloroquine resistance transporter gene* (*Pfcrt*) K76, and increased copy number of *Pfmdr1* are linked with parasite tolerance against lumefantrine (26, 31–33). However, other studies have shown that despite the high prevalence of these markers to a nearly fixation level, the cure rate of AL has remained acceptable (34–36). Conversely, mutants *Pfmdr1* 86Y, 184Y, and 1246Y and *Pfcrt* 72C, 73V, 74I, 75E, and 76T are associated with reduced *in vivo* efficacy of chloroquine and amodiaquine (26, 35). Point mutations in *P. falciparum plasmepsin* 2 and 3 (*Pfplasmepsin2/3*), and increased copy number of *Pfplasmepsin* 2 are also linked with resistance against piperaquine (37–39).

Sulfadoxine-pyrimethamine (SP) replaced chloroquine as first-line treatment for uncomplicated malaria in many malaria-endemic countries in early 1980s (40, 41). However, the widespread of *P. falciparum* resistance against SP led to the discontinuation of the drug as first-line in early 2000s. In Tanzania, SP was adopted as first-line in 2001, and it was withdrawn in 2006 (1). Currently, the World Health Organization (WHO) recommends SP for intermittent preventive treatment in pregnancy (IPTp) (42, 43). SP use for IPTp is safe and at its inception, it was sufficient to prevent the adverse consequences of malaria in pregnancy, including maternal anemia and low birth weight (42). However, the increase in *P. falciparum* resistance against the drug is threatening the future usefulness of IPTp-SP. *P. falciparum* parasite resistance against pyrimethamine is conferred by point mutations in the gene encoding for dihydrofolate reductase (*Pfdhfr*) at codons 50, 51, 59, 108, and 164 (44, 45), whereas that against sulfadoxine is conferred by dihydropteroate synthase (*Pfdhps*) at codons 436, 437, 540, 581, and 613 (44, 46). Mutation at *Pfdhfr* 164 is not common in Africa (44). The *Pfdhfr* and *Pfdhps* mutations are able to act synergistically to enhance the level of SP resistance. Quintuple mutant haplotype consisting of triple mutations of *Pfdhfr* (51I/59R/108N) and double mutations of *Pfdhps* (437G/540E) is associated with clinical and parasitological SP treatment failure (44, 47, 48). However, IPTp-SP remains efficacious and confers protection especially against maternal anemia, placental malaria, premature birth, and low birth weight even in settings with a high level of quintuple mutations (42, 47–49). Nonetheless, IPTp-SP efficacy for preventing maternal anemia and low birthweight is reduced when more than 10% of the *P. falciparum* parasites in a setting acquire an additional *Pfdhps* A581G mutation, leading to the sextuple mutant (*Pfdhfr* 51I + 59R + 108N/ *Pfdhps* 437G + 540E + 581G) (47, 50, 51). The WHO recommends the discontinuation of IPTp-SP in areas where the prevalence of *Pfdhps* mutant 540E (a proxy for quintuple *Pfdhfr-Pfdhps*) is more than 95% and that of 581G (a proxy for sextuple *Pfdhfr-Pfdhps*) has exceeded 10% (47, 52).

Thus, it is important to regularly monitor the spread of markers of artemisinin, partner drugs, and SP resistance in Tanzania. This will help to plan for the containment strategies early in the course of spread of the resistance against the drugs and prevent the increase in mortality and other malaria-related complications that would ensue as a consequence. The purpose of this study was therefore to assess the prevalence of markers of resistance against artemisinin, partner drugs, sulfadoxine, and pyrimethamine in Nanyumbu and Masasi Districts, Tanzania.

## MATERIALS AND METHODS

### Study area

**Fig 1:**
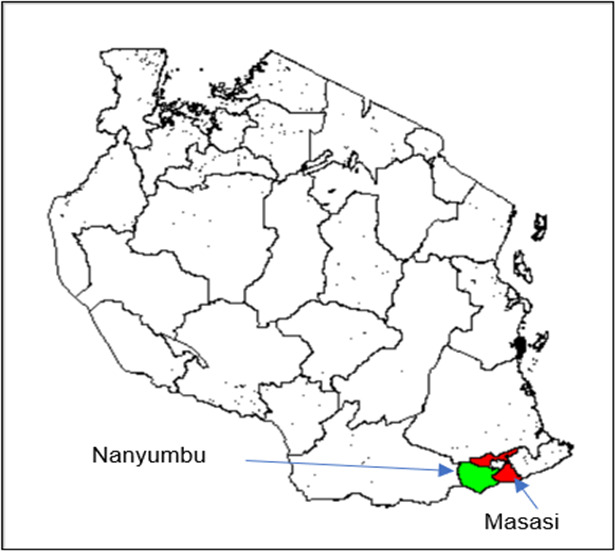
Map of Tanzania showing the study districts, Masasi and Nanyumbu (53).

This study was conducted in Nanyumbu and Masasi Districts, Mtwara Region, between July 2020 and August 2021. The region is in the South of Tanzania, with the two districts bordering Mozambique (Fig. 1). Subsistence farming, petty trade, and small-scale mining are the major economic activities of the population in both districts. Nanyumbu had 19 health facilities, while Masasi district had 40 facilities. The area has annual rainfall averaging 939 mm and average temperature of 25.4°C. The rainy season in the Districts is between January and April. Malaria transmission in both districts is high during the rainy season, peaking just after the end of the season. *P. falciparum* is the major malaria parasite. Insecticide-treated bed nets, diagnosis and treatment with ACT, and IPTp-SP constitute a backbone of malaria control measures in the Districts. AL is the first-line treatment for uncomplicated *P. falciparum* malaria in the Districts since 2006, and DHPQ an alternate first-line since 2016 (1–3).

### Study design and population

This was part of a large community-based cross-sectional malariometric survey conducted among children aged below 5 years in 20 wards selected randomly from the two Districts (54). In each ward, a centrally located health facility was determined, and the villages around the facility were identified. Thereafter, basic demographic information was obtained from the village leaders and health facilities to facilitate the enrollment of the participants.

Afebrile and febrile children regardless of their sexes were recruited in the survey. The participants were included if they were aged between 3 and 59 months, residents of the study area, and their caregivers consented for the children to be involved in the study. Participants were excluded if they had severe illness, had a history of using antimalarial drugs in the past 30 days or were under cotrimoxazole prophylaxis. A malariometric survey was conducted by collecting blood samples for determining the infection status, infecting *Plasmodium* species, number of infecting parasite strains, molecular markers of drug resistance, and hemoglobin levels.

### Procedures for data collection

Caregivers of eligible children were requested to bring their children to the catchment facilities where all the clinical and laboratory assessments were performed. At each ward, the survey was conducted for three consecutive days.

The clinical assessment involved taking a history of clinical symptoms, history of intake of antimalarial drugs in the past 30 days or cotrimoxazole prophylaxis use, and clinical examination including measurement of axillary temperature. Laboratory assessment involved collection of finger-prick blood samples for the detection of presence of *Plasmodium spp.* infection using malaria rapid diagnostic test (mRDT, SD Bioline Malaria Ag P.f/Pan), prepare thick smears for microscopy to determine density of asexual parasites, and thin smears for asexual *Plasmodium* species determination. The smears were prepared for all children. The samples were also used to measure hemoglobin concentration. Furthermore, the blood samples were blotted on 3 MM Whatman filter papers, air-dried at room temperature for 3–4 h, packed in individual plastic bags containing desiccant sachets, and then stored for molecular analysis of infecting *Plasmodium* species, number of strains of the infecting parasites, and markers of resistance. Thin smears were fixed using absolute methanol. Both thin and thick smears were stained using 3% Giemsa for 1 h and examined at 100 high power fields under immersion oil. Parasites on a thick smear were counted against 200 white blood cells (WBCs), and then multiplied by 40, assuming a milliliter of blood to have a WBC count of 8,000. A smear was considered negative if no parasite was seen after examining 100 fields. Two laboratory technicians read all the slides independent of one another. A third reading was requested in case of positive versus negative readings or a difference in parasite density of greater than 30%.

Hemoglobin concentration was measured using HemoCue Hb 301+ (HemoCue AB, Ängelholm, Sweden) spectrophotometer. A participant with a hemoglobin level <11 g/dL was considered anemic.

### Molecular analysis

The molecular analysis was performed by Wellcome Sanger Institute, UK. The genomic DNA was extracted from dried blood spots on filter paper using QIAmp DNA Investigator Kit (Qiagen, Valencia, CA, USA) and subsequently amplified by applying selective whole genome amplification (sWGA) as previously described by Oyola et al. (2016) (55). Amplicon sequencing using Illumina sequencing was performed following the SpotMalaria platform that contains markers for drug resistance, species identification as well as 101 SNP barcodes (56). For the *Pfk*13 gene, BTB/POZ and *Pfk*13-repeat propeller domains were analyzed and included all the validated and candidate markers published by the WHO in 2020 (57). Likewise, *Pfmdr1, Pfcrt, P. falciparum Exonuclease (PfEXO*)*, P. falciparum artemisinin-resistance genetic background* (*PfPGB*)*,* and *Pfplasmepsin2/3* genes were analyzed to assess markers of partner drugs resistance, and *Pfdhps and Pfdhfr* were analyzed for markers of SP resistance.

#### Species identification

*Plasmodium* species were determined by amplification and sequencing of two conserved regions flanking the 18S rRNA, the mitochondrial cytochrome b (*cytb*) and the apicoplast caseinolytic protease C gene (*clpC*) (58). The SNPs within these regions are able to differentiate the five species of *Plasmodium* (*P. falciparum, P. vivax, P. malariae, P. ovale, and P. knowlesi*). Briefly, a primary PCR amplification was followed by an additional amplification. PCR amplicons were purified using AmPure XP kit (Agencourt) (Roche Technical Bulletin No. 2011-007). Quality and purity of the amplicons were checked using the Agilent DNA 1000 assay kit on a 2100 Bioanalyzer (Agilent Technology) and subsequently quantified using the Quant-iT Picogreen dsDNA reagent (Invitrogen) on a Fluoroskan Ascent microplate Fluorometer (ThermoScientific). The amplicons were further processed following the GS Junior emPCR LibA method (Version April 2011) for emulsion PCR (emPCR) using a low copy per bead ratio (0.25 cpb). DNA-enriched beads were loaded onto a GS Junior Picotiter plate following the GS Junior sequencing manual (Version April 2011), and sequencing was performed in both forward and reverse direction using the GS Junior Titanium sequencing kit.

Thereafter, the *sff file* program (SFF Tools, Roche) was used to split raw sequence data based on multiplex identifier (MID). Low quality and short reads (<200 bp) were excluded prior to analysis. High-quality filtered reads were mapped to a reference database comprising 18S rRNA, *cytb,* and *clpC* gene sequences of *Plasmodium* spp. downloaded from GenBank. Unmapped sequences were analyzed using BLAST searches against the NCBI nucleotide database and the *Plasmodium* database (Plasmodb). *Plasmodium* species genotypes were identified by querying consensus sequences against the NCBI GenBank database.

#### Multiplicity of infection (MOI)

The estimation of the *P. falciparum* MOI from SNP genotyping data were performed using the programs COIL (59) and real MCOIL (60). The genotypes were determined at 101 SNP barcodes that have been chosen to analyze the diversity between parasites. The MOI was expressed as the estimated number of genetically distinct parasites within the infection from which the sample was taken.

#### Markers of drug resistance

Targets for genotyping were identified and multiplex PCR primers were designed using a modified version of the MPrimer software (61) and the exact design of the primer sequences can be obtained from the MalariaGEN protocols (62). Primers were designed to amplify products between 190 and 250  bp and were combined into three pools. A two-step protocol was used to first amplify the target regions of the parasite genome, followed by a second PCR to incorporate sequencing and multiplexing adapters. Batched samples were sequenced in a single MiSeq lane combining all PCR products. Samples were de-plexed using the multiplexing adapters and individual CRAM files were aligned to a modified amplicon *Pf*3D7 reference genome. Genotyping was done using bcf tools as well as custom scripts to filter and translate genotypes into drug resistance haplotypes. A Genetic Report Card containing detailed genetic data were generated to enable data analysis.

### Study outcomes

The outcome of interest was the prevalence of *P. falciparum* point mutations responsible for artemisinin, partner drugs, and SP resistance. Secondary outcomes include (i) species of the infecting parasites and their prevalence; (ii) prevalence of the parasites with multiple strains; and (iii) prevalence of haplotypes responsible for artemisinin, partner drugs, and SP resistance.

### Statistical analysis

Descriptive analysis was used to analyze the point mutations and haplotypes for *P. falciparum* responsible for artemisinin, partner drugs, and SP resistance; parasites with multiple strains and species of the infecting parasite. Categorical variables were summarized in cross-tabulations and reported as frequency with associated percentages. Continuous variables were summarized into means and reported with associated 95% confidence intervals (CIs) or standard deviations (SD) and minimum (min) and maximum (max) values.

## RESULTS

### Baseline characteristics of the study participants

A total of 531 blood samples were involved in this analysis for the infecting *Plasmodium* species, MOI, and molecular markers of drug resistance. These samples were a part of 2,340 total samples collected in a large study. The baseline characteristics of the 531 participants are presented in Table 1, whereas that of the total population involved in the large study has been presented elsewhere (54).

**TABLE 1:** Baseline characteristics of the study participants^*a*^

Variable			
Age in years, mean (SD), min, max	2.7 (1.3)	0.3	5.0
Sex, male (%)	271 (51.0)		
mRDT positive, n (%)	373 (70.2)		
Microscopy positive, n (%)	212 (39.9)		
Parasite density/μL of blood, geometric mean, 95% CI	368	256	530
Hemoglobin concentration, (SD), g/dL	10.3 (1.5)	5.8	14.7
Anemia (Hb <11 g/dL), n (%)	355 (66.9)		
Axillary temperature in °C, mean (SD), min, max	37.2 (1.0)	35.0	40.3
Febrile (body temperature ≥37.5°C), n (%)	195 (36.7)		

^*a*^ SD, standard deviation; °C, degree Celsius; CI, confidence interval.

### Infecting *Plasmodium* species and MOI

A total of 259 samples were successfully amplified for the infecting *Plasmodium* species. Of the analyzed parasites, 98.8% (256/259) were *P. falciparum*, 0.8% (2/259) were *P. malariae*, and 0.4% (1/259) was a mixed infection of *P. falciparum* and *P. ovale*. Infections with multiple strains were detected in 52.7% (119/226) of the participants. Of the infections with multiple strains, 66.4% (79/119), 14.3% (17/119), 8.4% (10/119), and 7.6% (9/119) had 2, 3, 4, and 5 MOI, respectively. The MOI of 6, 8, 10 and 14 occurred each in one participant.

### Prevalence of markers of drug resistance

#### Single nucleotide polymorphism

Markers assessed for artemisinin and partner drug resistance are presented in Table 2, and they included *Pfk13*, *Pfmdr1*, *Pfcrt*, *PfPGB*, *PfEXO*, and *PfPlasmepsin*2/3.

**TABLE 2:** Prevalence of markers of artemisinin and partner drug resistance^*a*^

Gene	Position, SNP, percentage (n/N)									
*Pfk13*	516D	0.9% (1/103)								
	578A	1.9% (2/103)								
	637V	3.9% (4/103)								
*Pf*crt	72C	100% (304/304)								
	74M	91.7% (276/301)	74I	0.7% (2/301)	74M/I	7.6% (23/301)				
	75N	91.9% (274/298)	75E	0.7% (2/298)	75N/K	0.3% (1/298)	75N/E	6.4% (19/298)	75N/D	0.7% (2/298)
	76K	91.0% (273/300)	76T	0.7% (2/300)	76K/T	8.3% (25/300)				
	93T	100% (290/290)								
	97H	100% (290/290)								
	218I	100% (254/254)								
	220A	95.9% (237/247)	220S	1.2% (3/247)	220A/S	2.8% (7/247)				
	271Q	97.6% (248/254)	271Q/E	2.4% (6/254)						
	333T	100% (250/250)								
	353G	100% (292/292)								
	371R	96.1% (324/337)	371I	0.6% (2/337)	371R/I	3.3% (11/337)				
*Pfmdr1*	86N	100% (185/185)								
	184Y	34.3% (48/140)	184F	40.0% (56/140)	184Y/F	25.7% (36/140)				
	1034S	100% (134/134)								
	1042N	100% (137/137)								
	1226F	100% (138/138)								
	1246D	100% (138/138)								
*PfEXO*	415E	100% (407/407)								
*PfPGB*	127V	100% (407/407)								
	128D	100% (397/397)								
	193D	100% (181/181)								
	326N	100% (265/265)								
	356I	99.3% (284/286)	356I/T	0.7% (2/286)						
	484T	100% (145/145)								
*Pfplasmepsin 2/3*		100% (53/53)								

^*a*^ Amino acids: A, Alanine; C, Cysteine; D, Aspartic acid; E, Glutamic acid; F, Phenylalanine: G, Glycine; H, Histidine; I, Isoleucine; K, Lysine; M, Methionine; N, Asparagine; Q, Glutamine; R, Arginine; S, Serine: T, Threonine: V, Valine; Y, Tyrosine. Differences in denominators are due to missing genotypes in some samples.

A total of 103 samples were successfully analyzed for Pfk13, and 93.2% (96/103) of them were wild type. Only seven samples had mutations, but none carried any of the validated Pfk13 markers. For the Pfcrt, the majority of the parasites carried the wild-type SNPs at codons 72, 74, 75, and 76. Likewise, at the additional codons 220, 271, and 371, the majority of the parasite isolates carried the wild-type SNPs.

For the *Pfmdr1*, the majority of the parasite isolates carried the wild-type N86, Y184, and D1246, which are responsible for mefloquine and lumefantrine susceptibility. Likewise, all the parasites carried wild-type SNPs at additional codons S1034, N1042, and F1226.

On the other hand, for the *Exonuclease* (*PfEXO*) gene, all the parasites carried the wild-type E415. For the artemisinin resistance genetic background (*PfPGB*) genes, including *Pfarps* at positions 127 and 128, *ferredoxin* at position 193, *Pfcrt* at positions 326, and *Pfmdr1* at position 484, the parasites selected for the wild type, except for *Pfcrt* at position 356, where 0.7% (2/286) of the parasites were a mixed infection of I/T. Furthermore, all the parasites were wildtype for *Pfplasmepsin* 2/3.

Markers assessed for pyrimethamine and sulfadoxine resistance included *P. falciparum* dihydrofolate reductase (Pfdhfr) and *P. falciparum* dihydropteroate synthase (Pfdhps), respectively, and are presented in Table 3. For the pyrimethamine marker Pfdhfr, at codons 51, 59, and 108, the majority of the parasites carried the mutant SNPs I, R, and N, respectively. However, all the parasites carried wild-type Pfdhfr 164. On the other hand, for the sulfadoxine marker Pfdhps, the majority of the parasites carried wild-type SNPs S, A, and A at codons 436, 581, and 613, respectively. At positions 437 and 540, mutant SNPs G and, respectively, were the majority.

**TABLE 3:** Prevalence of markers of sulfadoxine and pyrimethamine resistance^*a*^

Gene	Position, SNP, percentage (n/N)											
*Pfdhfr*	16A	100% (187/187)										
	51N	4.8% (9/188)	51I	86.2% (162/188)	51N/I	9.0% (17/188)						
	59R	89.2% (165/185)	59C	4.3% (8/185)	59C/R	5.9% (11/185)						
	108N	100% (163/163)										
	164I	100% (161/161)										
	306S	100% (182/182)										
*Pfdhps*	436S	90.7% (147/162)	436F	1.2% (2/162)	436A	1.2% (2/162)	436S/F	2.5% (4/162)	436S/C	0.6% (1/162)	436S/A	3.7%6/162
	437G	75.8% (125/165)	437A	11.5% (19/165)	437G/A	12.1% (20/165)						
	540K	12.8% (20/156)	540E	71.2% (111/156)	540K/E	16.0% (25/156)						
	581A	99.2% (130/131)	581G	0.7% (1/131)								
	613A	96.1% (124/129)	613S	1.6% (2/129)	613A/S	2.3% (3/129)						

^*a*^ Differences in denominators are due to missing genotypes in some samples.

#### Haplotypes

Table 4 presents the haplotypes assessed for *Pfcrt*, *Pfmdr1*, *Pfdhfr*, and *Pfdhps* markers. For *Pfcrt*, most of the parasites were carrying CVMNK haplotype. On the other hand, for *Pfmdr1*, the majority of the parasites were carrying NFD haplotype, followed by the wild-type haplotype NYD. The rest were different mixed infections.

**TABLE 4:** Prevalence of haplotypes associated with artemisinin, partner drugs, sulfadoxine, and pyrimethamine resistance

*Pf*crt *N* = 305 (%)		*Pfmdr1* *N* = 198 (%)		*Pfdhfr* *N* = 153 (%)		*Pfdhps* *N* = 117	
CVMNK	271 (88.9)	NFD	48 (24.2)	**IRN**I	119 (77.8)	S**GE**AA	78 (66.7)
CV(M/I)(N/E)(K/T)	19 (6.2)	NYD	39 (19.7)	N**RN**I	8 (5.2)	SAKAA	12 (10.3)
CVMN(K/T)	2 (0.7)	N[Y/F]D	32 (16.2)	**I**C**N**I	6 (3.9)	**A**AKAA	2 (1.7)
CVM(N/K)(K/T)	1 (0.3)	–^*a*^	–	(N/I)RNI	12 (7.8)	**F**AK**GS**	1 (0.9)
CV(M/I)(N/D)K	1 (0.3)	–	–	I(C/R)NI	7 (4.6)	**F**AKA**S**	1 (0.9)
CV(M/I)(N/D)(K/T)	1 (0.3)	–	–	N(C/R)NI	1 (0.7)	S(G/A)(K/E)AA	6 (5.1)
CVIET	2 (0.7)	–	–	I(R/H)NI	1 (0.7)	SG(K/E)AA	5 (4.3)
–	–	–	–	(N/I)(C/R)NI	1 (0.7)	(S/A)(G/A)(K/E)AA	5 (4.3)
–	–	–	–	–	–	(S/F)(G/A)(K/E)A(A/S)	2 (1.8)
–	–	–	–	–	–	SA(K/E)AA	1 (0.9)
–	–	–	–	–	–	S(G/A)KAA	1 (0.9)
–	–	–	–	–	–	S(G/A)EAA	1 (0.9)
–	–	–	–	–	–	SGEA(A/S)	1 (0.9)
–	–	–	–	–	–	(S/C)(G/A)(K/E)AA	1 (0.9)
–	–	–	–	–	–	(S/A)AKAA	1 (0.9)

^*a*^ –, not applicable.

In the *Pfdhfr*, the triple mutation **IRN**I occurred in more than a three-quarter of the parasites. Double mutations N**RN**I and **I**C**N**I occurred in 5.2% (8/153) and 3.9% (6/153) of the parasites, respectively. None of the parasites carried the wild-type NCSI haplotype. For the *Pfdhps* marker, triple mutations **F**AK**GS** occurred in less than 1% of the parasites, whereas double mutations S**GE**AA and **F**AKA**S** occurred in 66.7% (78/117) and 0.9% (1/117) of the parasites, respectively. Only 10% of the parasites were carrying the wildtype SAKAA haplotype.

Quadruple mutations **I**C**N**I-S**GE**AA, **IRN**I-**A**AKAA, and N**RN**I-S**GE**AA occurred in very few parasite isolates. Quintuple mutation **IRN**I-S**GE**AA occurred in more than half of the isolates, and sextuple mutation **IRN**I-**F**AK**GS** occurred in less than 1% of the parasites.

## DISCUSSION

The ACT and SP play a significant role in the treatment and prevention of malaria in sub-Saharan Africa. However, the spread of partial artemisinin resistance may derail the gains made in the fight against malaria. This study aimed to assess the prevalence of markers of resistance associated with artemisinin and its partner drugs, sulfadoxine, and pyrimethamine in Masasi and Nanyumbu Districts. In these districts, none of the parasites carried any of the validated *Pfk13* markers of artemisinin resistance. Nonetheless, a small proportion of the parasites carried non-validated *Pfk13* 516D, 578A, and 637V, and the linkage of these markers with ACTs’ efficacy has not been demonstrated. Furthermore, nearly all of the parasites carried the wild-type *PfPGB* (a group of artemisinin-resistance genetic background markers) except *Pfcrt* 356 (I/T), which occurred in 0.7% of the parasites. ACT has remained highly efficacious in Masasi and Nanyumbu Districts (9). However, recent studies conducted between 2019 and 2023 have shown the presence of the validated *Pfk13* 561H in five regions, including Pwani, Kagera, Tabora, Njombe, and Manyara (21–23), with the highest prevalence occurring in Kagera region (22, 23). In Kagera, *Pfk13* 561H was associated with delayed parasite clearance but not with ACT treatment failure (23). Containment programs should be initiated to contain the spread of artemisinin resistance and hence protect the partner drugs. Tanzania is among 10 countries with the highest prevalence of malaria globally (63); therefore, the spread of artemisinin resistance and consequently the failure of the partner drugs will be catastrophic. Contrarily, the overall prevalence of *Pfcrt* (C72, M74, N75, and K76) and *Pfmdr1* (N86, F184, and D1246) was high, with *Pfcrt* C72, and *Pfmdr1* N86 and D1246 reaching 100%. Previous studies in Tanzania and other countries have also indicated the high prevalence of *Pfmdr1* N86 and *Pfcrt* K76 of above 90% following years of widescale use of AL (4, 33, 35, 64, 65). Wild-type *Pfcrt* K76 and *Pfmdr1* N86, Y184, and D1246 have been associated with parasite tolerance against lumefantrine (26, 31–33, 64). However, despite the prevalence of these markers reaching the saturation point, AL has remained effective for the treatment of malaria in Tanzania and other African countries (4, 35, 36, 64, 65). This may probably indicate that the drug has multiple sites of action and the detected mutations are not specific for the partner drug. Few additional *Pfcrt* mutations were detected at codons 220 (220S and 220A/S), 271 (271Q/E), and 371 (371I and 371R/I); however, their effect on the efficacy of the ACTs has not been demonstrated. Furthermore, *PfEXO* and *Pfplasmepsin* 2/3 point mutations, and increased copy number of *Pfplasmepsin* 2 have been associated with parasite resistance against piperaquine (37–39). In this study, all the isolates were wild-type *PfEXO* and *Pfplasmepsin* 2/3. Nonetheless, a previous study conducted in Tanzania in 2017 indicated the presence of increased copy number of *Pfplasmepsin* 2, although the efficacy of DHPQ remained very high (6). On the other hand, the wild-type *Pfcrt* CVMNK haplotype was the most prevalent, accounting for 88.6%. This implies that the majority of parasites may be sensitive to chloroquine. Nonetheless, the mutant *Pfcrt* CVIET haplotype was found in 0.7% of the isolates and was all from Likokona ward, Nanyumbu district. CVIET haplotype is associated with chloroquine resistance (26, 66). This haplotype may expand rapidly if chloroquine is reintroduced in the area. Mutant *Pfmdr1* NFD haplotype was the most prevalent in the area, followed by the wild-type NYD haplotype. Similar findings have been reported in Tanzania in previous studies (22, 33). The mutant NFD haplotype is linked with tolerance against lumefantrine (33).

On the other hand, more than 80% of the parasites carried the mutant *Pfdhfr* 51I, 59R, and 108N. Other studies have found the prevalence of the triple mutations to be near fixation (22, 67). However, for the sulfadoxine resistance marker *Pfdhps* 437G and 540E, the two markers essential for the elevation of SP resistance were found in more than 70% of the parasites. Ninety percent of the remaining assessed *Pfdhps* codons selected for the wild type. Unlike in this study, near fixation prevalence of 437G and 540E has been reported in a previous study in Tanzania (22). Furthermore, triple *Pfdhfr* mutation **IRN**I haplotype was the most prevalent, followed by double mutations N**RN**I and **I**C**N**I haplotypes. Similar to our findings, previous studies in other regions in Tanzania (67, 68), and other countries, including the Republic of Congo (82%), Ghana (81%) (45), and Nigeria (93.8%) (69), have indicated the predominance of *Pfdhfr* triple mutant **IRN**I. The *Pfdhfr* double mutant **I**C**N**I was dominant in Kenya (45). None of the parasites carried the wild-type NCSI haplotype. Likewise, for the *Pfdhps*, majority of the parasites were carrying double mutation S**GE**AA. Similar findings have been reported in other studies (67). However, unlike in a previous study in Muheza, Tanga, in this study, no parasite was carrying the triple S**GEG**A mutation (67). Only 10% of the parasites in this study were carrying the wild-type SAKAA. A similar low prevalence of the wild-type *Pfdhps* SAKAA has been reported in the previous study (67). Quadruple mutations occurred only in 8% of the parasites, and the majority of them carried ICNI-S**GE**AA. More than half of the parasites were carrying quintuple mutation **IRN**I-S**GE**AA. A previous study in other six regions of Tanzania found this quintuple mutation to be the most prevalent (68). Quintuple mutation is responsible for resistance against SP (44, 47, 48), but the drug has remained effective for IPTp (43, 47–49). Sextuple mutation (**IRN**I-**F**AK**GS)** occurred only in one participant. Sextuple mutation is associated with the loss of IPTp-SP efficacy, and it is characterized by higher risks of infections in peripheral blood and placental blood and higher parasite densities (47, 50). With this very low prevalence of sextuple mutation in Masasi and Nanyumbu Districts, the IPTp-SP remains effective. On the other hand, the drug pressure is associated with mutation of the parasite against the drug. However, it is not clear why the prevalence of quintuple (*Pfdhfr* 51I + 59R + 108N/*Pfdhps* 437G + 540E) and sextuple *Pfdhfr*51I + 59R + 108N/*Pfdhps* 437G + 540E + 581G) mutants in Masasi and Nanyumbu Districts, Tanzania, where IPTp-SP is still in use, was significantly lower than that reported in Rwanda where SP use as first-line treatment and for IPTp was stopped more than 15 years ago (70).

Nearly 99% of the infecting parasites were *P. falciparum*. Only two participants were infected with *P. malariae*, and one participant had a mixed infection of *P. falciparum* and *P. ovale*. Previous studies have indicated a higher prevalence of non-*falciparum* malaria (71–73). Both *P. falciparum* and *P. malariae* infections can be cleared by ACT; however, *P. vivax* and *P. ovale* have a tendency to form hypnozoites, a dormant stage that occurs in the liver. Hypnozoites cannot be cleared by ACT and are responsible for the relapse of the infection. Therefore, there is a need to adopt and use primaquine. Primaquine is potent against hypnozoites and can therefore prevent relapse. On the other hand, more than half of the infections had multiple strains. Likewise, two-thirds of the infections with multiple strains were comprised of two strains, and the remaining one-third comprised three strains and above. The MOI is normally used to indicate the intensity of malaria transmission in a setting. In this study, the presence of high prevalence of multiple strains clearly indicates that Masasi and Nanyumbu Districts remain a moderate to high transmission setting despite two decades of scale-up of malaria interventions in the area. Therefore, there is probably a need to integrate novel tools to the existing toolbox to reduce and possibly eliminate the infection in the Districts.

On the other hand, the study had limitations, including the overall low rate of amplification of the genes and the variation in depth of sequencing between genes. These limitations might have been attributed to the low quality of the samples.

### Conclusion

No parasite carried any of the validated markers of partial artemisinin resistance; however, the prevalence of resistance markers for partner drugs, particularly *Pfcrt* and *Pfmdr1,* reached the saturation point. Sextuple *Pfdhfr-Pfdhps* mutations occurred only in one patient; therefore, SP remains efficacious for IPTp in the Districts.

## Data Availability

All relevant data are within the manuscript and its supplemental material.

## SUPPLEMENTAL MATERIAL

Data S1 aac.01751-24-s0001.xlsx Age and sex of the participants, and the markers of drug resistance analyses.

ASM does not own the copyrights to Supplemental Material that may be linked to, or accessed through, an article. The authors have granted ASM a non-exclusive, world-wide license to publish the Supplemental Material files. Please contact the corresponding author directly for reuse.
